# Accuracy of Artificial Intelligence in Predicting Facial Changes Post-Orthognathic Surgery: A Comprehensive Scoping Review

**DOI:** 10.4317/jced.61500

**Published:** 2024-05-01

**Authors:** Asim A. Almarhoumi

**Affiliations:** 1 M.Orth RCSEd. Division of Orthodontics, Department of Preventive Dental Sciences, College of Dentistry and Dental Hospital at Taibah University, Madinah, Saudi Arabia

## Abstract

**Background:**

Accurate prediction of facial soft tissue changes post-orthognathic surgery is crucial for treatment planning and patient communication. Current models pose limitations due to the complexity of facial biomechanics and individual variances. Artificial intelligence (AI) has emerged as an important tool in many disciplines, including the dental field.

**Objectives:**

The aim of this scoping review is to assess the accuracy of AI in predicting facial changes post-orthognathic surgery in comparison to traditional models. Explore the strengths and limitations of the current AI models.

**Material and Methods:**

Following PRISMA-DTA guidelines, a comprehensive search was conducted manually and through Medline, Embase, Web of Science, Scopus, and Google Scholar databases was conducted, focusing on studies that applied AI models with various machine learning and deep learning algorithms for post-surgical outcome prediction. Selection criteria were based on the PICO format, emphasizing studies that compared AI-predicted outcomes with actual post-surgical results. Literature was searched until January 31, 2024.

**Results:**

The initial search result yielded 1579 records. After screening and assessment for eligibility, seven studies met the inclusion criteria, with publication dates ranging from 2009 to 2023. Several AI algorithms were evaluated on different orthognathic surgical procedures, revealing the high predictive accuracy of AI models across various facial regions.

**Conclusions:**

AI demonstrates significant potential for enhancing the precision of facial outcome predictions following orthognathic surgery. However, despite the promising results, limitations such as small sample sizes and a lack of external validation were noted. Further research with larger, more diverse datasets and standardized validation methods is essential for optimizing AI’s clinical utility.

** Key words:**Artificial Intelligence (AI), Machine Learning (ML), Deep Learning (DL), Orthognathic Surgery, Facial Soft-tissue Prediction, Predictive Accuracy, Orthodontics.

## Introduction

Artificial Intelligence (AI), defined as “a system’s ability to interpret external data correctly, to learn from such data, and to use those learnings to achieve specific goals and tasks through flexible adaptation.” has had a significant impact since it was established in the 1950s (1). Initially focused on creating “thinking machines” that mimic human intelligence and behaviour, AI has evolved to encompass a variety of technologies capable of replicating human decision-making and problem-solving abilities (2). This innovation in technological progress is practical, enhancing human productivity by efficiently completing tasks using extensive datasets to convert data into actionable information for specific tasks, such as the diagnostic processes in medical sciences, improving accuracy in diagnosis and patient care outcomes (3).

**Figure 1:**
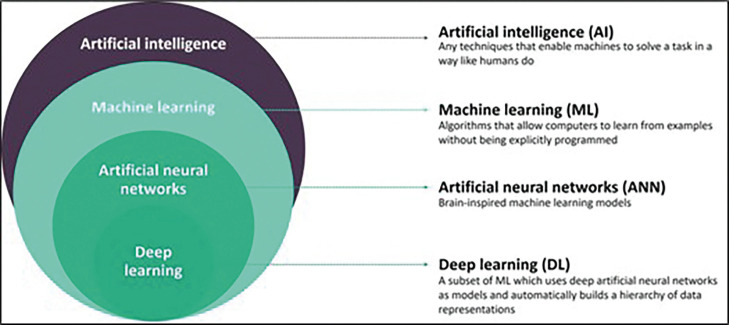
Infographic representation of the relationship between artificial intelligence (AI), machine learning (ML), artificial neural networks (ANNs), and deep learning (DL). Obtained from source: (38).

Machine Learning (ML) is an integral subset of AI, incorporating algorithms that improve with exposure to more data. Deep Learning (DL), a subcategory of ML, uses neural networks to estimate complex non-linear associations between input and output variables (Fig. 1). These algorithms, capable of accomplishing tasks at a faster pace than humans, mark a significant advancement in AI (4). Deep learning applications, including image recognition, speech recognition, and natural language processing, demonstrate the efficiency of these algorithms. Deep learning has shown remarkable performance in computer vision tasks, and their application extends to various fields including healthcare, where they assist in diagnosis and decision-making processes (5,6).

**Figure 2:**
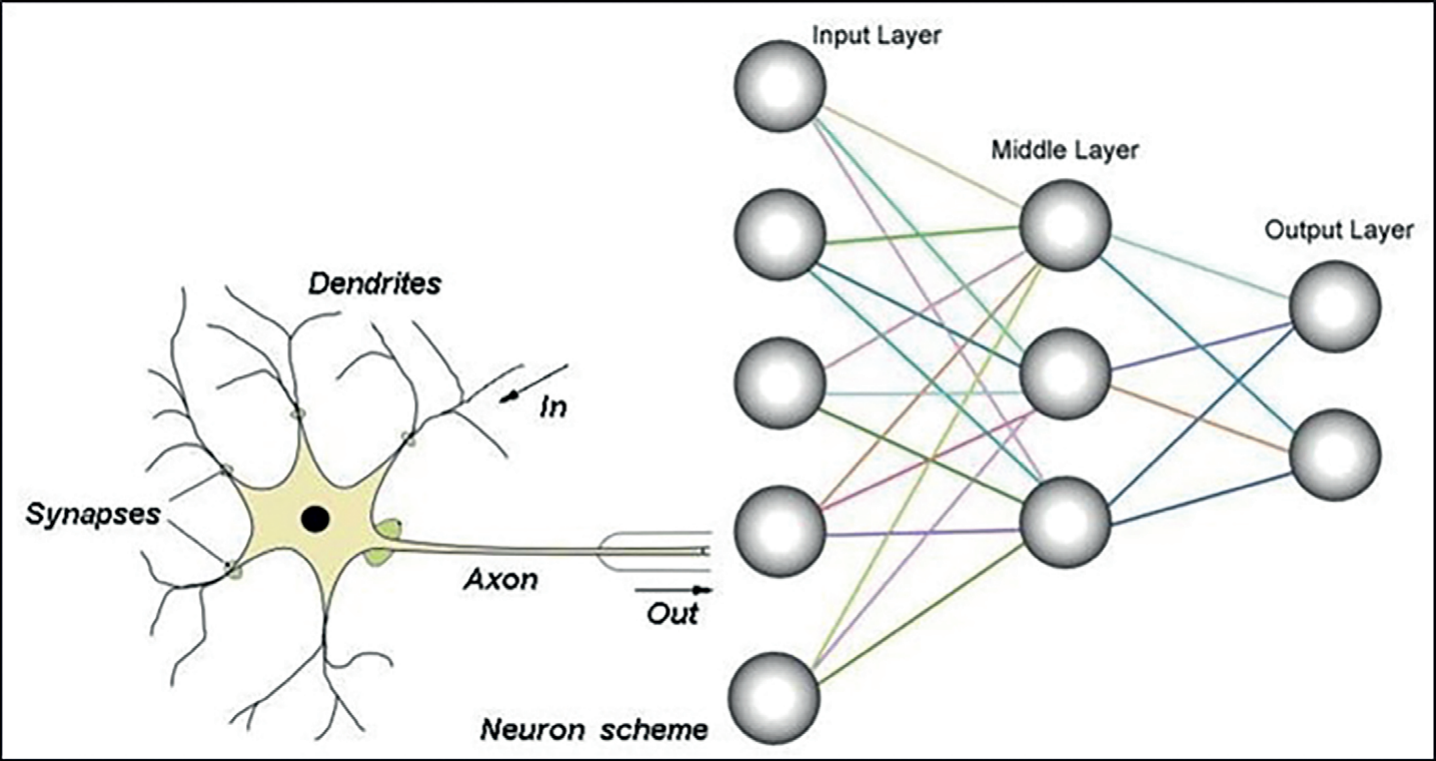
Schematic illustration of biological neuron (left) and a simple artificial neural network (ANN) (right). Obtained from source: (39).

Artificial Neural Networks (ANN) and Convolutional Neural Networks (CNN) are subsets of DL. ANNs are inspired by the biological neural networks in the human brain and play a key role in machine learning, enabling the analysis of complex relationships within large datasets. Typically, an ANN is structured with at least three layers: an input layer, an output layer, and one or more hidden layers. These layers are interconnected, forming a network that processes information (Fig. 2) (7,8).

**Figure 3:**
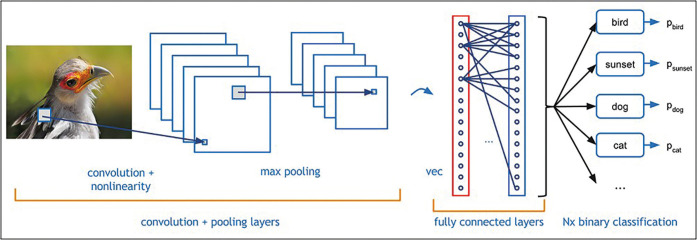
Schematic diagram for the image classification process of convolutional neural networks (CNNs). Obtained from source: (40).

CNNs, known for their exceptional performance in handling high-resolution images, are vital for deep learning. They are especially suited for image and pattern recognition tasks, thanks to their convolutional layers, pooling layers, and fully connected layers. Convolutional layers apply filters to input data to create feature maps, ideal for recognizing objects, shapes, and patterns. Pooling layers simplify the feature maps by preserving essential information but reducing their size, which helps in making the network more efficient. Finally, the fully connected layers integrate these insights for better decision-making, making CNNs superior to ANNs for tasks involving images (Fig. 3) (7,9).

The integration of AI technologies, including Machine Learning ML and Deep Learning DL, into healthcare, specifically in fields like dentistry and orthodontics, represents a significant advancement. They have become increasingly prevalent due to their exceptional accuracy in handling large data, learning tasks, predictions and decision-making processes. This advancement has allowed them to match the performance of skilled healthcare professionals in various aspects of patient care. Notably, the application of ANNs and CNNs within orthodontics exemplifies this progress. Initially used for radiographic analysis such as radiographic lesions detection and automatic cephalometric landmarks identification (10-12). These technologies have evolved into more complex decision-making tools for treatment planning, showcasing their versatility and effectiveness in enhancing clinical outcomes (13-16).

In orthodontics, enhancing facial aesthetics is a key goal. Improving facial aesthetics is a primary motivation for patients undergoing orthognathic surgery, with the prediction of irreversible outcomes presenting significant challenges. However, accurately predicting post-treatment facial appearance is challenging due to complex biomechanics and minimal craniofacial changes. Individual factors like healing processes, bone structure, and soft tissue response, which are difficult to accurately predict, play a crucial role in these outcomes (17-20).

The acknowledged AI’s capabilities are not to be overlooked. Furthermore, it could be an invaluable tool for precisely predicting the post-surgical facial appearance following extensive orthognathic structural changes. There is a lack of thorough reviews on the effectiveness of AI and its various models in predicting facial topology post-orthognathic surgery. Therefore, the objective of this scoping review is to systematically examine the literature on the precision of AI’s capabilities in predicting changes in facial soft tissue following orthognathic surgeries. 

## Material and Methods

-Search strategy:

This structured scoping review was conducted following the guidelines outlined in the Preferred Reporting Items for Systematic Reviews and Meta-Analyses extension for Diagnostic Test Accuracy (PRISMA-DTA) (21). A comprehensive search was conducted in Medline via Pubmed, Embase, Web of Science, Scopus and Google Scholar electronic databases to identify and select the literature for this paper. The retrieved results encompassed all indexed literature within each database until January 31st, 2024, as no specific date range was specified.

The search strategy in this review was conducted in line with the (Participant/Population, Intervention, Control/Comparison and Outcome/Result) (PICO) format; Patients undergoing orthognathic or cranio-facial surgery was identified as the population (P). Intervention (I) was identified as the use of AI including different ML and DL algorithms for the prediction of post-surgical soft-tissue outcome. Control (C) classical clinical-based or computer-based outcome prediction without AI involvement. The Outcome (O) the level of diagnostic test accuracy, sensitivity and specificity between AI-predicted and post-surgical obtained actual soft-tissue outcomes.

The relevant MeSH (Medical Subject Headings) terms used for the search strategy were:

1- “Artificial Intelligence”(MeSH)

2- “Machine Learning”(MeSH)

3- 1 OR 2

4- “Orthognathic Surgical Procedures”(MeSH)

5- “Treatment Oucome”(MeSH)

6- “Prediction”(MeSH)

7- 5 OR 6

8- 3 AND 4 and 7

The search syntax using keywords with Booleans and truncations for each database searched is found in ( Table 1). Furthermore, a manual search was conducted complementing the electronic search process, involving the examination and exploration of the reference lists of the initially chosen articles.

-Criteria for literature eligibility:

Only original clinical studies that followed the designated PICO format were included in this review (randomised- and/or non-randomised clinical trials, longitudinal prospective or retrospective cohort clinical studies). Attempts were made to contact corresponding authors for any relevant inaccessible literature, such as ‘abstract only’ or missing full-text to obtain the full-text wherever possible. Any other literature such as Case reports, correspondence letters, commentaries, reviews were excluded. 

## Results

**Figure 4:**
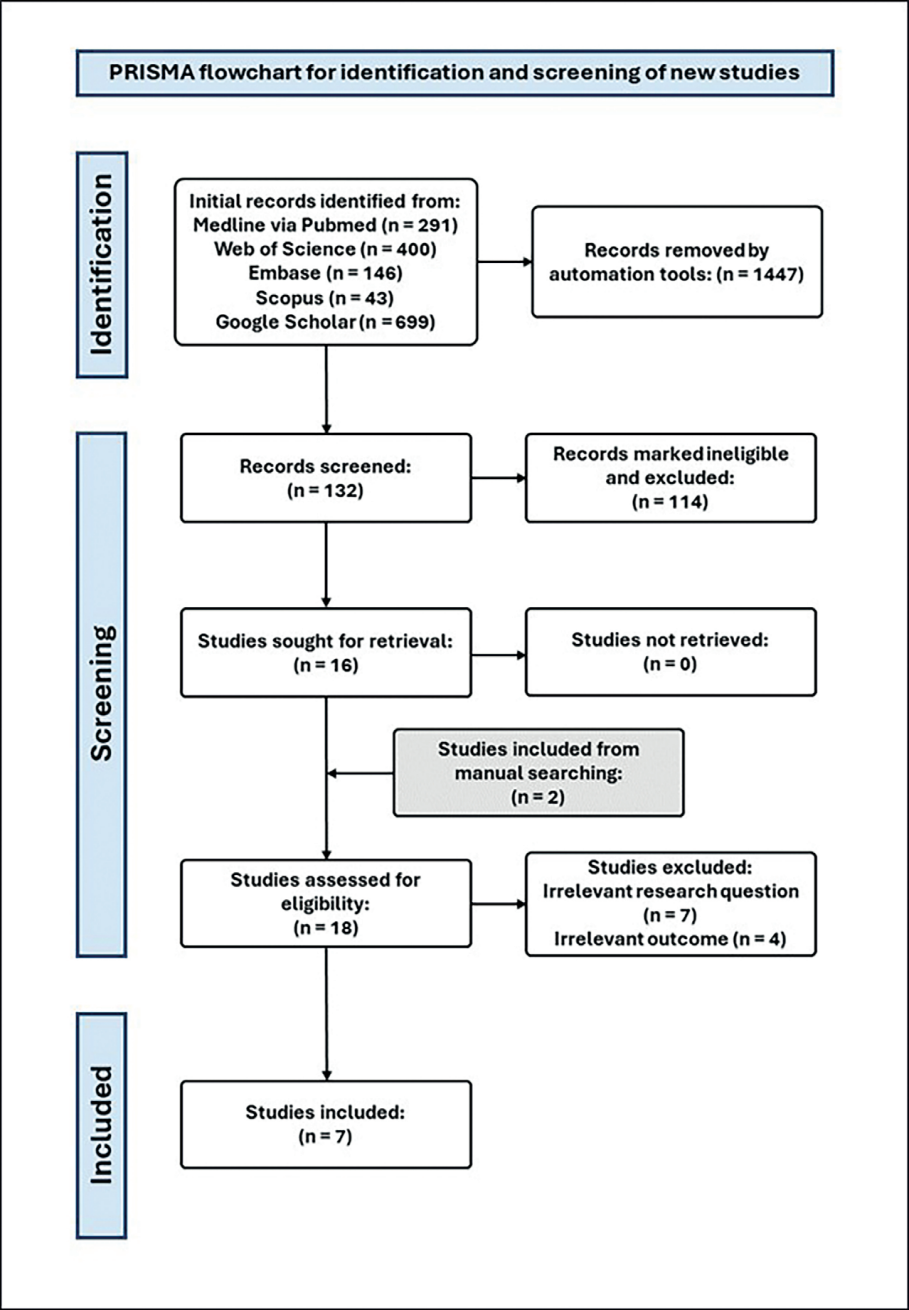
Preferred Reporting Items for Systematic Reviews and Meta-Analyses (PRISMA) flowchart.

-Search strategy results:

A comprehensive search of all databases and a manual search yielded the identification of 1579 articles. Following the implementation of the exclusion criteria and duplicates detection, the total of 132 studies were retained. Out of the total, 125 studies were eliminated following a thorough assessment of the title and/or abstract’s relevance to the scope of this review. A total of seven studies were ultimately incorporated into the current review and subjected to data extraction (Fig. 4).

-Characteristics of the included studies:

Seven studies that have utilized the use of AI models in predicting facial soft tissue outcomes following orthognathic surgery were included in this scoping review, as indicated in ( Table 2). The outcomes primarily assessed the precision of soft tissue prediction, highlighting mean errors for different facial areas and overall accuracy rates of prediction. All studies were retrospective by design. Three were case-control studies (22-24); similar were cohort studies (25-27); and one was an experimental proof-of-concept study design (28). The sample sizes within the studies ranged from five to 137 patients. Three studies reported surgical intervention types such as bimaxillary surgery, mandibular advancement, and maxillary advancement surgeries (22,24,26), while four studies did not (25,27-29). AI models like Artificial Neural Networks (ANN) and machine learning methods like Logistic Regression (LR), Ridge Regression (RR), and Least Absolute Shrinkage and Selection Operator (LASSO) are used in the articles. So are deep learning (DL) models like convolutional neural networks (CNN), CNN-based deep spatial multiband VGG-NET, and more specialized architectures like autoencoder neural networks, PointNet++, and FSC-Network. The imaging modalities employed were lateral cephalograms and facial photographs in two studies, CT and CBCT scans in three studies, and three more studies employed more sophisticated techniques such as virtually constructed 3D photos.

## Discussion

Implementation of AI and DL in Post-orthognathic Facial Soft-tissue Prediction:

Soft tissue response prediction has been the subject of extensive research, with the mass-spring model (MSM), finite element model (FEM), and mass tensor model (MTM) being the most prevalent. These models serve as the basis for the vast majority of Visualized Treatment Objective (VTO) software packages presently employed in clinical practice. When viewed as a whole, the accuracy of these software applications appears to be clinically satisfactory. However, these historically used software, based on the assumption that facial profile adapts to dental structures at a fixed ratio, has faced doubts due to prediction errors, particularly within the lower facial third and the lips (30,31). Research indicated that facial morphology is affected by a multitude of factors, including age, gender, and dental issues, suggesting a need for personalized prediction models (21,30-32).

To overcome the limitations of legacy models, Lu *et al*. first proposed using Artificial Intelligence via supervised ANNs to enhance post-orthognathic surgery image predictions. Their trained ANN model’s accuracy was compared with Dolphin Imaging software for predicting postsurgical cephalograms and the generated soft tissue outline video images. The study showed a significant accuracy increase, especially for the soft-tissue pogonion, with 70% and 100% of predictions within <1mm and <2mm error ranges, respectively. The lower lip saw 70% and 80% accuracy improvements within <1mm and <2mm, respectively, marking a noTable enhancement from original predictions. However, the subnasale region was still challenging, with 50% of predictions exceeding a 2mm error margin. Overall, 80% of predictions were within a <2mm error range, demonstrating a substantial improvement in AI’s forecasting of post-surgical outcomes (22).

Knoops *et al*. conducted a study in which they used a 3D morphable model (3DMM) to automatically categorize orthognathic surgery candidates and compared several machine learning algorithms to assess the accuracy of the generated face simulations for orthognathic surgery treatment plans. Least-angle regression (LARS) and ridge regression (RR) was found to have the lowest average error (1.1 ± 0.3 mm), followed by least absolute shrinkage and selection operator regression (LASSO) (1.3 ± 0.3 mm) and linear regression (LR) (3.0 ± 1.2 mm). These accuracies are equivalent to conventional computer-assisted surgical planning techniques. This ML predictions has a sensitivity of 95.5%, specificity of 95.2% for classification of the need for orthognathic surgery, and an average accuracy of 1.1 ± 0.3 mm for post-surgical soft-tissue prediction. Furthermore, positive and negative predictive values were 87.5% and 98.3%, respectively. Moreover, they compared the AI-predicted faces to the mean global face and mean bespoke postoperative face to ensure patient-specific predictions. Results showed a smaller difference between AI prediction and postoperative 3D scan (1.1 mm) compared to the mean global face (1.8 mm) and the mean bespoke postoperative face (1.6 mm) (25).

Tanikawa *et al*. proposed using landmark-based geometric morphometric methods (GMMs) and deep learning to predict 3-D facial topography after orthognathic surgery, leveraging recent computational advances. GMMs utilize homologous landmarks on biological specimens in developmental biology to categorize individuals within a common morphospace. Their study achieved a post-surgical facial topography prediction accuracy of 74% within <1mm and 100% within <2mm. The AI system they proposed for predicting facial form after orthognathic surgery demonstrated a mean error of 0.94 ± 0.43 mm, with observed maximum errors ranging from 0.8 to 1.2 mm at the nasal ala, chin, and corners of the mouth, which was considered clinically accepTable. However, there was no direct comparison between their AI model and any existing software used in orthodontic surgery, such as Dolphin Imaging, Mimics, and others (26).

Ter Horst *et al*. compared the Autoencoder deep neural network with the Mass Tensor Model (MTM) for predicting soft tissue profiles after an average of 5 mm mandibular advancement surgery. DL-based predictions were found to be more accurate than those of the MTM, resulting in lower error rates and more precise soft-tissue predictions. The Autoencoder algorithm showed a mean absolute error (MAE) range of 1.0 to 1.4 mm across various facial regions, including the lower face, lower lip, and chin. Conversely, the Mass Tensor Model (MTM) algorithm had a MAE range of 1.5 to 2.0 mm for the same areas. Furthermore, the prediction accuracy within a >1mm range was significantly higher for the DL model (42.9% to 64.3%) compared to the MTM model (14.3% to 21.4%). The study also employed Root Mean Squared Error (RMSE) to quantify the average magnitude of errors in soft tissue simulations. RMSE operates in two-fold. Firstly, it highlights large errors, thereby illuminating significant discrepancies between predictions and actual outcomes. Secondly, it treats overprediction (positive values) and underprediction (negative values) equally, preventing the issue where positive and negative values cancel each other out. However, reported limitations included a bias towards a more female distribution in the AI test set and deviations in the AI model’s ability to handle cases with significant facial asymmetry (24).

An advanced deep learning (DL) model was introduced by Ali *et al*. The Deep Spatial Multiband VGG-NET CNN model, an improved VGG-NET architecture, which is able to process complex spatial correlations and different data bandwidths in facial images. It was trained and validated on 313,318 head CT scans and medical records from multiple institutions, with 21,095 scans for algorithm training and 491 for clinical validation. The approach outperforms previous AI models (SEMI-AUTOMATIC, UNET, 3D-UNET)(33) in post-surgical outcome prediction with accuracy, sensitivity, and specificity rates of 93.7%, 99.9%, and 99.8%. notably, it was evaluated with Dice and Jaccard scores, which allowed for a more sophisticated assessment than MAE (27).

Lampen *et al*. developed a deep learning model for predicting facial tissue deformation in orthognathic surgery. The method uses the PointNet++ architecture, which allows for fast and accurate prediction of soft-tissue deformation. The network accepts detailed facial meshes and explicit boundary types, reducing simulation time while maintaining accuracy comparable to the finite element method (FEM). The model demonstrated high accuracy and compatibility with complex facial structures, with mean errors between 0.159 and 0.642 mm across subjects. The inclusion of explicit boundary types improved simulations with large deformations. This approach significantly reduces simulation time compared to traditional FEM methods, providing rapid feedback for surgical planning. However, the study had some design limitations, being a proof-of-concept test on only five subjects and a lack of direct comparison with existing methods. It also yielded mixed results in performance across different scenarios. The inclusion of explicit boundary types improved performance in large deformation scenarios but decreased in small ones, suggesting inconsistent benefits across different scenarios. The study’s limitations also suggest that the model’s effectiveness may not be universally applicable across different scenarios (28).

Ma *et al*. developed FSC-Net, a deep learning system for planning orthognathic surgery. This advanced network can convert preoperative face forms into postoperative predictions by analyzing planned adjustments in the underlying bone structure. It functions under a weakly supervised learning model, achieving faster outcomes than the state-of-the-art biomechanical computation, finite element modelling with realistic lip sliding effect (FEM-RLSE), by a factor of 15 as it only required 2 minutes to complete the computations compared to 30 minutes of laborious work by an experienced surgeon using the FEM-RLSE method. It can predict facial results using low-resolution CBCT images without requiring high-quality meshes, with mean errors ranging from 2.85 to 3.08 mm. Despite its rapid and accurate simulations, FSC-Net faces challenges with lip region predictions and rare or extensive deformities (29).

-Strengths And Limitations

Studies included in this review consistently demonstrated that AI and DL models offer at least similar if not an enhanced accuracy compared to traditional prediction methods. This improved accuracy in treatment objective visualization is crucial for patient communication and realistic expectation management. Furthermore, these models significantly reduce computation times, enabling rapid feedback and multiple simulations, thereby enhancing clinical workflows and patient experience. Additionally, the application of AI and DL in surgical planning provides a cost-effective alternative to conventional treatment simulations, potentially leading to better patient outcomes (34,35).

On the other hand, it is not without limitations, and they must be addressed to fully harness the potential of AI and DL. The reported studies demonstrated high variability in AI models’ algorithms, which can lead to inconsistent results. This emphasizes the need for standardization in model development and validation processes. Small sample sizes in many studies limit the generalizability of findings, necessitating efforts to increase sample size and dataset diversity to improve model robustness. Additionally, the lack of external validity raises concerns regarding the performance of models on datasets different from the training data. Cross-validation and external validation are needed to be certain about the reliability and generalizability of the AI predictive accuracy. Moreover, the scarcity of big data for AI training and validation is a significant challenge that warrants the need for collaborative efforts to compile comprehensive databases for robust training and validation (36,37).

## Conclusions

In conclusion, AI holds great promise for improving the prediction of facial soft-tissue changes following orthognathic surgery. While current models have demonstrated a high level of accuracy and reliability, ongoing research and development are essential to overcome existing limitations and fully realize the potential of AI in this field. By addressing these challenges, AI can become an invaluable tool for clinicians, enabling more precise and personalized treatment planning and ultimately improving patient outcomes.

## Figures and Tables

**Table 1:** Search strategy used for electronic databases.

Database	Results	Keywords
Medline via PubMed	291	(artificial intelligence OR machine learning OR deep learning OR neural network* OR algorithm* OR machine intelligence OR ANN OR CNN) AND (orthognathic OR orthognathic surgery OR jaw surgery OR mandibul* OR maxill* OR facial OR cranio*) AND (prediction OR simulation OR outcome OR plan OR assessment OR forecast) AND (soft-tissue OR facial)
Web of Science	400	((ALL= (artificial intelligence OR machine learning OR deep learning OR neural network* OR algorithm* OR machine intelligence OR ANN OR CNN)) AND ALL= (orthognathic OR orthognathic surgery OR jaw surgery OR mandibul* OR maxill* OR facial OR cranio*)) AND ALL=(prediction OR simulation OR outcome OR plan OR assessment OR forecast) AND ALL=(soft-tissue OR facial)
Embase	146	TX (artificial intelligence or machine learning or deep learning or neural network or ANN or CNN) AND TX (prediction or outcome or simulation or forecast) AND TX (orthognathic or mandibl* or maxill* or cranio*) AND TX (soft-tissue or facial)
Scopus	43	TX (artificial intelligence or machine learning or deep learning or neural network or ANN or CNN) AND TX (prediction or outcome or simulation) AND TX (orthognathic or mandibl* or maxill* or cranio*) AND TX (soft-tissue or facial)
Google Scholar	699	(artificial intelligence OR machine learning OR deep learning OR neural network* OR algorithm* OR machine intelligence OR ANN OR CNN) AND (orthognathic OR orthognathic surgery OR jaw surgery OR mandibul* OR maxill* OR facial) AND (prediction OR simulation OR outcome OR plan OR assessment) AND (soft-tissue OR facial)

**Table 2:** Characterestics of the included studies.

Date	Author	Study Design	AI Model	Sample Size	Groups	Imaging	Surgery	Outcomes
2009	Lu et al. (22)	Retrospective case control	ANN	30 patients with bimaxillary protrusion	AI-improved prediction: Training set: 20 patients. Test: 10 patients. Comparison group: Dolphin Imaging VTO Control: Post-operative ground-truth images.	Lateral cephalograms and profile photographs.	Bimaxillary surgery	Accuracy rate within mean error <2mm = 84.5%. sensitivity, specificity not reported
2019	Knoops et al. (25)	Retrospective cohort	Global model ML: 3DMM Soft-tissue prediction ML: LR RR LARS LASSO	ML (3DMM) training: 4261 faces. Post-surgical Soft-tissue prediction: 113 patients.	Training/Test: 80-20%	3D morphable models obtained via virtual cameras.	Not specified	Accuracy 1.1±0.3 mm, Sensitivity: 95.5%, Specificity: 95.2%; Mean Error (mm): LR (3.0±1.2) RR (1.1±0.3) LARS (1.1±0.3) LASSO (1.3±0.3)
2021	Tanikawa, Yamashiro (26)	Retrospective cohort	DL (CNN) Model S: Orthognathic patients.	Total: 137 patients Orthognathic surgery: 72 patients.	11-fold cross-validation: Training set: 10 sets. Test set: 1 set. Detailed numbers were not reported.	Lateral cephalograms and 3D facial images.	Maxillary advancement and Mandibular setback	Mean Error (mm): 0.89±0.3 Accuracy rate within mean error <1mm = 74% and <2mm = 100% Sensitivity and specificity not reported
2021	Ter Horst et al. (24)	Retrospective case control	Autoencoder neural network	133 subjects	AI group: Training set: 119 Test set: 14 Comparison group: Mass Tensor Model.	3D photos, CBCT	Mandibular advancement	Mean absolute error: Lower face 1.0±0.6 mm; Accuracy rate within mean error <1mm = 64.3% and <2mm = 92.9%. sensitivity, specificity not reported
2022	Ali et al. (27)	Retrospective cohort	Deep spatial Multiband VGG-NET CNN	Developed by 21095 scans Sample size for outcome prediction not reported.	Training set: 80% Test set: 20%	CT	Not specified	Accuracy: 93.7%, Sensitivity: 99.9%, Specificity: 99.8%; Mean error not reported
2022	Lampen et al. (28)	Proof-of-Concept	PointNet++	5 subjects	Training/Validation/Test: 70-10-20%	Not specified	Not specified	Mean error between 0.159 and 0.642 mm; Accuracy, sensitivity, specificity not reported
2023	Ma et al. (23)	Retrospective case control	FSC-Net	40 patients	AI group: 5-fold cross-validation (8 patients per group): Training set: 4-fold. Test set: 1-fold. Comparison group: (FEM-RLSE)	CT	Not specified	Mean absolute error: Jaw region 3.08±0.8, Midface 2.85±0.74, Whole face 2.95±061. Accuracy, sensitivity, specificity not reported. No significant difference between the two methods. Speed improvement: <2 mins for simulation.

## Data Availability

The datasets used and/or analyzed during the current study are available from the corresponding author.
